# Supplementary material for: Suppression of the ABCA1 Cholesterol Transporter Impairs the Growth and Migration of Epithelial Ovarian Cancer
Source: Cancers (Basel). 2022 Apr 8;14(8):1878. doi: 10.3390/cancers14081878 (PMC9029800; doi:10.3390/cancers14081878)
Supplement: Supplementary file 1 [file cancers-14-01878-s001.zip › cancers-1625793-supplementary.pdf]

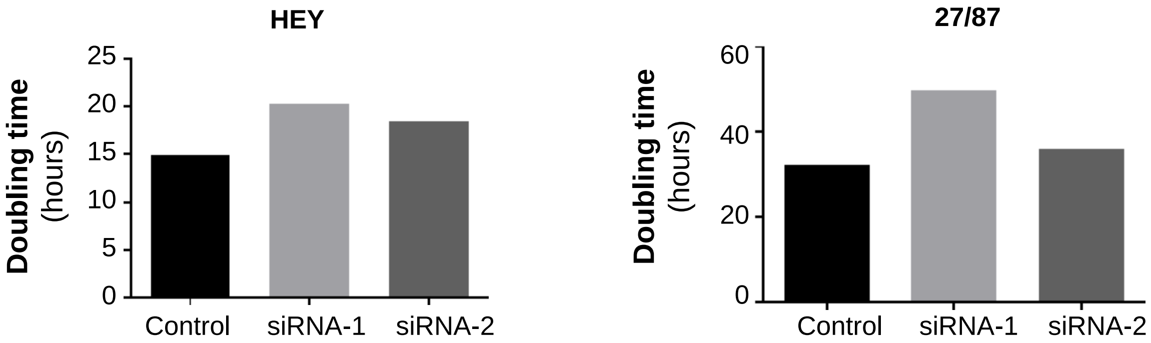

Figure S1: Doubling time of HEY and 27/87 EOC cells. Cell doubling times were extrapolated from growth curves performed over 72 hours.

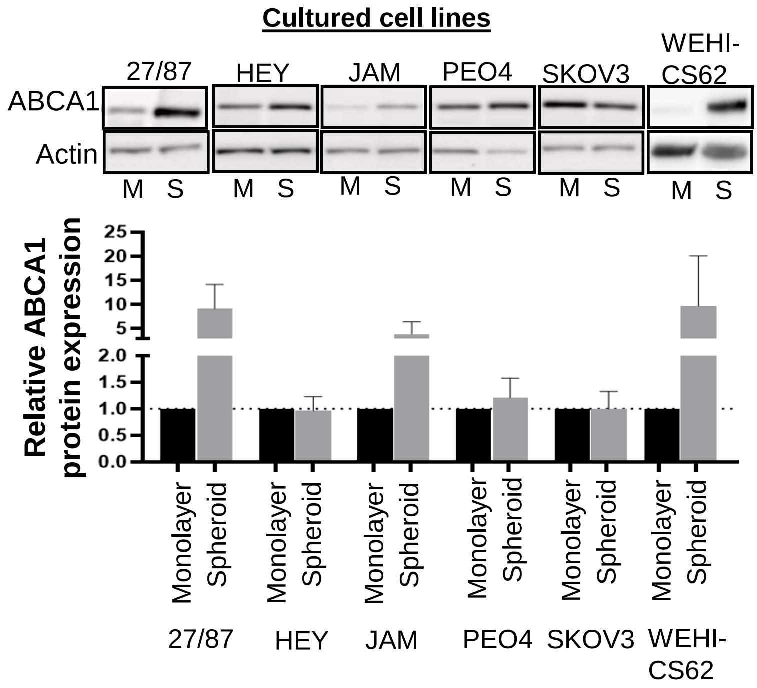

Figure S2: Western blots and respective densitometry analysis (column graphs) of ABCA1 protein expression in various EOC cell lines grown under monolayer or spheroid culture conditions. Protein levels in spheroids were expressed relative to monolayer culture for each cell line.

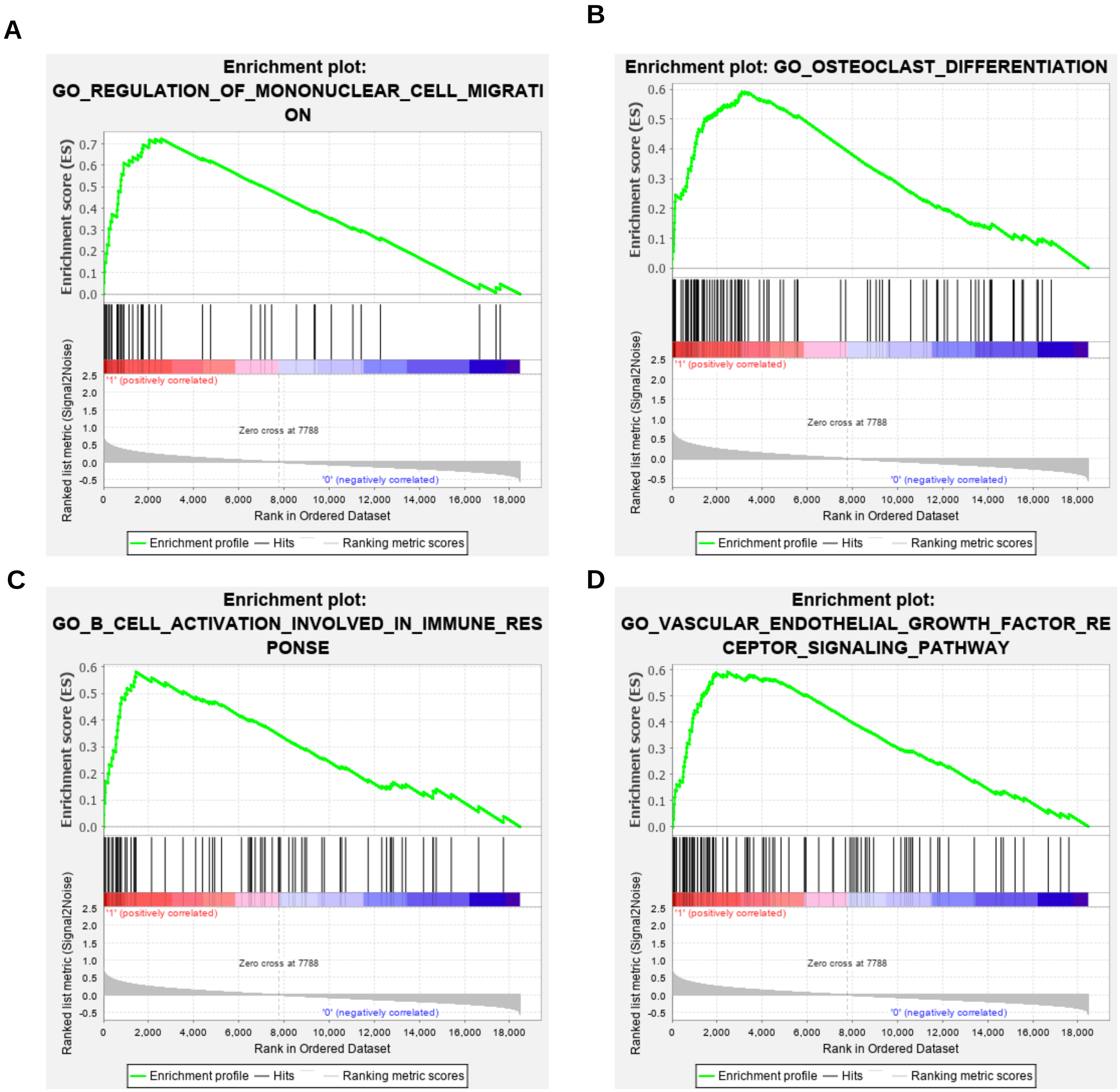

Figure S3: Additional gene set enrichment analysis (GSEA) pathways that are enriched in EOC tumors with high ABCA1 expression in epithelial ovarian cancer. (A) GSEA showing enrichment in the Gene Ontology (GO) gene set involved in mononuclear cell migration. (B) GSEA showing enrichment in the GO gene set involved in osteoclast differentiation. (C) GSEA showing enrichment in the GO gene set involved in immune response. (D) GSEA showing enrichment in the GO gene set involved in VEGFR signaling. False discovery rate (FDR) of <0.1 for all. N=498.

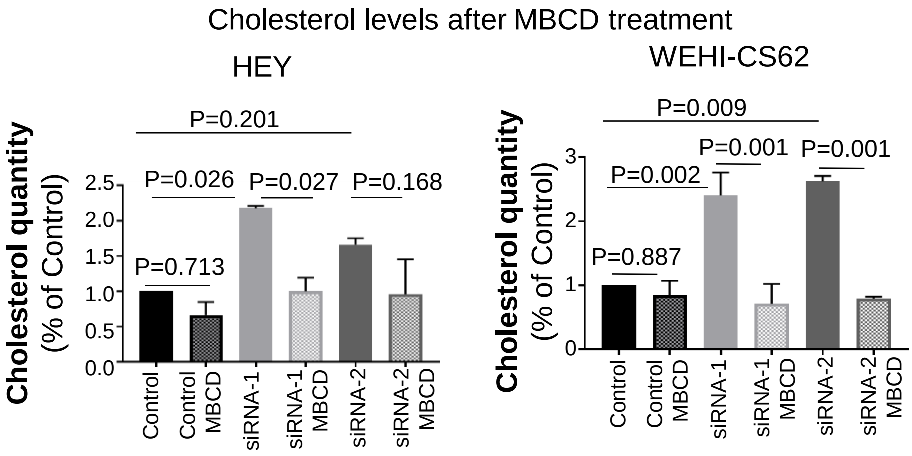

Figure S4: Quantification of intracellular cholesterol levels demonstrate the ability of methyl-B-cyclodextrin (MBCD) to offset the cholesterol accumulation caused by ABCA1 suppression. N=2. P-values were derived from two-way ANOVA. Results present mean ± SD.

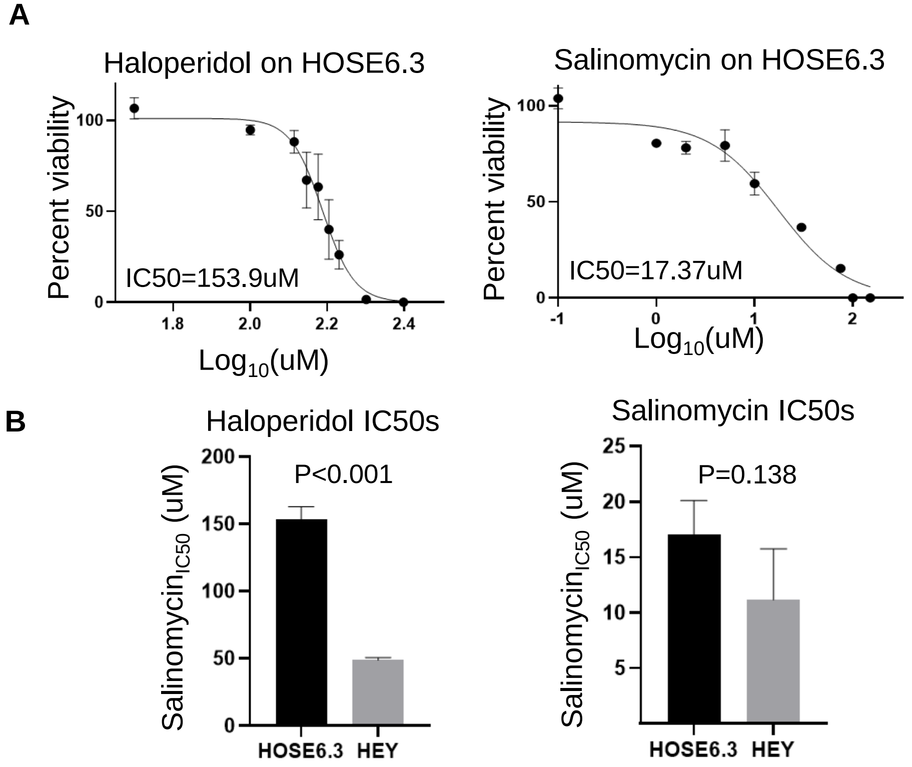

Figure S5: The differential effects of haloperidol and salinomycin on malignant HEY and non-malignant human ovarian surface epithelial (HOSE6.3) cells after 72 hours of treatment. (A) Resazurin-based cell viability assay assessing HOSE6.3 cells treated with the indicated drugs. (B) Comparison of the IC50 the drugs on HOSE6.3 versus HEY cells. Results represent mean ± SD. P-values were derived from unpaired t-test.
